# Online Prescribing of Sildanefil (Viagra) on the World Wide Web

**DOI:** 10.2196/jmir.1.2.e10

**Published:** 1999-12-31

**Authors:** Gunther Eysenbach

**Keywords:** Internet, Referral and Consultation, Fees, Pharmaceutical, Prescriptions, Drug, Commerce, Physician's Practice Patterns, Impotence, Piperazines, Medical History Taking, Quality of Health Care

## Abstract

**Background:**

A growing number of prescription medicines such as Viagra® are offered and sold directly to consumers on the Internet. Little is known about the structure and "quality" of these "virtual pharmacies" in terms of how responsibly "online-prescriptions" are actually issued.

**Objective:**

To determine to what extent Viagra is sold on the Internet despite clear contraindications.

**Methods:**

The World Wide Web was searched for companies who offer to issue prescriptions for Viagra online or sell Viagra without prescription. We pretended to be a patient in which the ordered drug (Viagra) is clearly contraindicated, and tried to obtain an online prescription for this drug on the Internet. Our test case was as a 69-year-old woman giving a sexual history of having "no orgasm," with obesity (165cm/78kg), coronary artery disease, and hypertension, and taking captopril, pravachol, atenolol, and erythromycin.

**Results:**

Twenty-two distinct companies were identified, consisting of three different types: 2 required a written prescription by a "real" physician, 9 dispensed the drug without any prescription at all, and 11 issued an "online prescription" after an alleged physician reviewed the online order form containing medical questions. We tested 10 of the latter type, among them 8 based in the USA. We ordered a total of 66 pills worth US$ 1,802.84. Three companies, among them both European companies, delivered within 6, 10, and 34 days respectively, despite Viagra being clearly contraindicated. In 80% no complete history was taken, in 70% inappropriate medical terminology was used, and in only 2 cases was the order form reviewed by a physician who identified himself.

**Conclusions:**

Although a surprisingly high number of Internet pharmacies declined delivery, the public should be alerted to the risks involved with prescription drug prescribing and dispensing via the Internet.

## Introduction

The World Wide Web has become a medium to advertise and dispense medicines directly to consumers. Since little is known about the structure and "quality" of these "virtual pharmacies" in terms of how responsibly "online-prescriptions" are actually issued, we created a fictitious patient for whom the ordered drug (sildanefil, known by the brand name Viagra®) was clearly contraindicated and tried to purchase this drug on the Internet.

## Methods

In March 1999, we searched for "cyberpharmacies" selling Viagra by entering the terms "+order +viagra" into a search engine (http://www.altavista.com). We reviewed the first 60 of the 60,000 pages found, and researched the company name and address behind the Internet addresses using InterNIC's "Whois" service [1]. Twenty-two distinct companies of three different types were identified: 2 required a written prescription by a "real" physician, 9 dispensed the drug without any prescription at all, and 11 issued an "online prescription" after an alleged physician reviewed the online order form containing medical questions answered by the patient. We tested 10 pharmacies of the latter type, among them 8 companies based in the USA, by posing as a 69-year-old woman giving a sexual history of having "no orgasm," with obesity (165cm/78kg), coronary artery disease, and hypertension, and taking captopril, pravachol, atenolol, and erythromycin. In this case, prescription of sildanefil would clearly not be indicated, as Viagra is not approved for females, and special caution has to be taken with patients having a history of cardiovascular disease [2]; besides, the fictitious patient was taking multiple medicines which could interact with sildanefil. We ordered a total of 66 pills among the 10 companies. The average price was US$ 17.00 per pill, ranging from US$ 8.33 to 50.00. Eight companies charged a consultation fee, ranging from US$ 65.00 to 89.00 (mean US$ 74. 88). In all cases, the consultation fee was only charged if the doctor determined that Viagra was "appropriate." Including shipping fees (US$ 34.00 on average), the total value of all orders was US$ 1,802.84.

## Results

An overview of all cyberpharmacies contacted is given in Table 1.

**Table 1:** Cyberpharmacies. Reasons for non-delivery: F=not indicated in females, I=import restrictions, M=medical reasons, X=no reason given

	***URL***	***Country of origin***	***Remarks***	***Total price***	***Delivered yes/no***
1.	http://kwikmed.com/	USA	Did not ask for concomitant medications. Affiliate program.	US$ 216.00 (10x50mg)	No/F - "cannot prescribe to females at this time"
2.	http://www.viagraguys.com/	USA	Offers to ship cimetidine (Tagamet®) with sildanefil to enhance its effect.	US$ 130.00 (3x50mg)	No/F - "even though there is no reason to believe that Viagra might be harmful to women ...in order to remain consistent with prescribing information...we are not filling prescriptions for women at this time."
3.	http://www.qualitymed.com/ http://www.viagracafe.com/	USA	Affiliate program.	US$ 196.00 (3x50mg)	No/F - "we know of its benefits in both sexes, however, we are unable to fulfill any orders for females at this time"
4.	http://www.viagra.nu/	Gibraltar (delivered from USA)	Very short medical history questionnaire.	US$ 59.80 (2 x 100mg)	Yes - (34 days)
5.	http://www.mdbyphone.com/	USA	Charged credit card but did not deliver.	US$ 149.00 (5 x 100mg)	No/I - "we are prohibited to deliver into Germany"
6.	http://thepillbox.com/ http://www.medicalcenter.net/	USA		US$ 236.00 (10 x 100mg)	No/I - "due to your country's import restrictions, we are unable to ship into your country"
7.	http://cyber.global-rx.com/	USA	Advertised as "miracle drug."	US$ 228.00 (10 x 50 mg)	No/X - "we must decline your order at this time."
8.	http://viagra.stiverson.com/	USA	Charged credit card but did not deliver.	US$ 194.00 (10 x 50mg)	No/M - " the doctor was concerned about your heart conditions and the medication you are on."
9.	http://www.net-dr.com/	USA	German questionnaire, prescription by US doctor. Sent email warning to stop other drugs.	US$ 219.00 (10 x 100mg)	Yes (10 days)
10.	http://swisspharma.com/	Switzerland (delivered from Spain)		US$ 99.70 (3 x 100mg)	Yes (6 days)

All companies requested that customers waive the site's liability in the event that they experienced health problems. While all sites asked whether customers were currently taking nitrates, only 4 specifically asked about recent "myocardial infarction," and only 2 of those used the lay term "heart attack." Seven asked about hypertension, but only 3 used the lay term "high blood pressure." Only 3 asked about retinitis pigmentosa, another important contraindication of sildanefil.

One site offered to ship, together with Viagra, tablets of cimetidine (Tagamet®), as it causes "56% increase in plasma sildanefil concentrations when coadministered with Viagra. This would indicate that increased effectiveness would be noted with the same dose of Viagra taken with 800mg of Cimetidine."

Three companies, among them both European companies, delivered within 6, 10, and 34 days respectively. One company sent an email warning to discontinue all other 5 medications when taking Viagra. A photocopy of the original Pfizer package insert was enclosed in 1 case, in all other cases incomplete package information was provided.

Two companies declined to deliver because of import restrictions, 1 declined to deliver without giving specific reasons, 3 declined to deliver because the drug is not approved for women (2 of them however claimed that they "know of the benefits for women"), and 1 did not deliver "because the doctor was concerned about your heart conditions and the medication you are on." Two of the companies which did not deliver nevertheless charged the credit card. The name of the consulting doctor was revealed in only 2 cases.

## Discussion

Although a surprisingly high number of Internet pharmacies declined delivery, the public should be alerted to the risks involved with prescription drug prescribing and dispensing via the Internet. In 30% of our cases, "prescriptions" were issued although clear contraindications existed, in 80% no complete history was taken, in 70% inappropriate medical terminology was used, and in only two cases was the order form reviewed by a physician who identified himself. Pharmacies claiming to "consult" consumers may be harmful, as the clients may rely on a non-existent "physician." However, even if doctors appear to be employed at the pharmacies, this is no guarantee for a safe drug-shopping experience for patients; in 2 out of the 3 cases where Viagra was delivered, physicians appeared to have approved the prescription, recalling the problem of the questionable credentials of "cyberdocs" [3]. Consumers should also be aware that Viagra on the Internet costs on average twice as much as in regular pharmacies, and that in our test 20% of the pharmacies charged the credit card without delivering a product. We estimate that at least 4,500-15,000 web pages offer online ordering of Viagra. Many dispensing companies run "affiliate" programs offering a commission to individuals who advertise and link customers to them [4], which is one explanation for the immense number of doorway pages, and another reason for incomplete and misleading drug information on the Internet. Future legislative action may target this lay-advertising practice. Consumer education and an international modus operandi for managing drug sales on the Internet are further steps that should be taken by the World Health Organization (WHO) [5] and other organizations.
